# SA physiotherapy: Moving forward with innovation in rehabilitation and the National Health Insurance Scheme

**DOI:** 10.4102/sajp.v80i1.2138

**Published:** 2024-11-07

**Authors:** Witness Mudzi

**Affiliations:** 1 School of Therapeutic Sciences, University of the Witwatersrand, Johannesburg, South Africa

In recent years, the field of rehabilitation has witnessed remarkable advancements driven by innovative technologies that are reshaping the way we approach recovery and health promotion. These innovations are enhancing the effectiveness of rehabilitation and making it more accessible and personalised.

Physiotherapy in South Africa has not been left behind. It is also evolving at an exciting pace, driven by a wave of innovation in rehabilitation practices, and the ongoing rollout of the National Health Insurance (NHI) system will add impetus to the process. As the country seeks to bridge healthcare disparities and improve access to quality services, physiotherapy should stand at the forefront of this transformation. With a renewed focus on technology, patient-centred care and integration within broader healthcare systems, South African physiotherapists are poised to impact the nation’s health and well-being.

## Innovation in rehabilitation: A new frontier

The surge in technological advances in physiotherapy has created new opportunities for innovative rehabilitation methods. Virtual reality (VR), telehealth and wearable technology have become valuable tools for physiotherapists. These innovations allow professionals to monitor and engage patients remotely, expanding access to care, especially in rural and underserved areas. For example, VR-based rehabilitation can simulate environments that help patients regain mobility and balance in a controlled and safe space. At the same time, wearable devices can provide real-time patient progress data, allowing for personalised interventions (Mlenzana 2020). Patients can receive guidance and support from physiotherapists through video calls, making rehabilitation services more accessible, especially in remote areas (Winstein & Requejo 2015). Artificial intelligence (AI)-powered rehabilitation tools such as VR systems, wearable devices and mobile apps can be used for immersive rehabilitation exercises, helping patients recover motor skills in a controlled, engaging environment, monitor patients’ physical activity and provide real-time feedback to both patients and healthcare providers, ensuring exercises are performed correctly and progress is tracked accurately (Winstein & Requejo 2015). The coronavirus disease 2019 (COVID-19) showed us that this area is primed for growth if properly managed.

The move towards evidence-based practice has also gained momentum, with physiotherapists increasingly adopting data-driven treatment planning and assessment approaches. South African universities and research institutions are investing in studies that explore the effectiveness of these new technologies and techniques, providing a solid foundation for their integration into clinical practice (Department of Higher Education and Training 2022).

## National Health Insurance: An opportunity for integration

The NHI system presents both challenges and opportunities for physiotherapy in South Africa. With its aim of providing equitable healthcare access for all citizens, the NHI emphasises the importance of multidisciplinary collaboration, prevention and early intervention, all areas where physiotherapy can make a significant contribution (National Department of Health 2019). However, for physiotherapists to play a central role in this new healthcare landscape, they must adapt to a system that prioritises efficiency and outcomes-based care. There is a need to adapt from the usual treatment-based approach to a more preventative-based one, meaning we must embrace our pivotal role as primary healthcare workers.

Under the NHI, physiotherapists must demonstrate their value as rehabilitation specialists and essential players in preventative care and chronic disease management. This shift calls for a broader understanding of physiotherapy beyond traditional musculoskeletal treatments. As defined by the South African (SA) Society of Physiotherapy, physiotherapists will increasingly need to engage with patients in holistic, community-centred approaches, focussing on promoting movement, reducing the risk of injury and managing long-term conditions such as arthritis, cardiovascular diseases and diabetes (SA Society of Physiotherapy 2021).

To align with the goals of the NHI, physiotherapists must also navigate integrating their services within public health structures. This involves building stronger relationships with other healthcare professionals and ensuring that physiotherapy is accessible in all settings, from primary care clinics to hospitals. By embedding physiotherapy services throughout the healthcare system, the NHI can improve rehabilitation outcomes and reduce the burden on tertiary care facilities (Motaze, Tumbo & Mbombo 2021).

## Challenges and the path forward

While the outlook is promising, the path forward is not without obstacles. One of the most significant challenges lies in training and retaining skilled physiotherapists in the public sector. Many professionals prefer private practice for better remuneration and working conditions (Louw, Morris & Grimmer 2018). The NHI must address these disparities by offering competitive contracting salaries, offering opportunities for professional growth, and support for continuous education to attract and retain talent within the public healthcare system.

Another critical challenge is ensuring that technological innovations reach the areas that need them most. While urban centres may have access to cutting-edge equipment and telehealth infrastructure, rural and remote areas often lag behind. Bridging this gap requires investment in digital infrastructure and targeted training programmes for physiotherapists working in underserved communities (Van Staden, 2021).

Another challenge is the ethical use of innovative technologies, particularly AI. Establishing governance frameworks to ensure AI is used ethically and responsibly and addressing issues like data privacy and bias are a must.

Despite these challenges, the future of physiotherapy in South Africa is bright. The profession’s ability to adapt and innovate is a testament to its resilience and dedication to improving patient care. As physiotherapists continue to embrace new technologies and integrate them into the NHI framework, they have the potential to transform the rehabilitation landscape, making quality care accessible to all South Africans.

## Embracing the future

To move forward effectively, South African physiotherapists must adopt a forward-thinking mindset, staying abreast of global trends while tailoring solutions to the local context. Innovation, collaboration and a commitment to patient-centred care will be the pillars of success. By embracing these principles, the physiotherapy profession can play a central role in South Africa’s healthcare evolution, contributing to a healthier and more resilient nation for future generations. The use of AI is pivotal in this process. Artificial intelligence can help manage patient records and rehabilitation data, ensuring accurate and up-to-date information is available to healthcare providers (Archer & Ellis 2024). Artificial intelligence can revolutionise rehabilitation by enabling more accurate diagnosis, personalised treatment plans and predictive analytics. Artificial intelligence-powered tools can analyse vast amounts of data to identify patterns and predict outcomes, helping therapists tailor interventions to each patient’s unique needs (Archer & Ellis 2024). This personalised approach enhances the effectiveness of rehabilitation and accelerates recovery. Integrating these AI-driven innovations allows the NHI scheme to provide more effective, efficient and accessible rehabilitation services, ultimately improving patient outcomes and reducing healthcare costs.

## Conclusion

Integrating these innovative technologies into rehabilitation practices is transforming the field, offering new hope and improved patient outcomes. As we continue to advance, we must ensure that these technologies are accessible, user-friendly and supported by robust evidence to maximise their potential benefits.

Incorporating AI and innovation into rehabilitation within the new NHI scheme can significantly enhance patient care, improve outcomes and streamline processes.

## References

[CIT0001] Archer, K.R. & Ellis, T.D., 2024, ‘Advances in rehabilitation technology to transform health’, *Physical Therapy* 104(2), pzae008. 10.1093/ptj/pzae008 38329471

[CIT0002] Department of Higher Education and Training, 2022, *National plan for higher education*, Department of Higher Education and Training, Pretoria.

[CIT0004] Louw, Q., Morris, L. & Grimmer, K., 2018, ‘Professional satisfaction and the migration of South African physiotherapists’, *Physiotherapy Research International* 23(1), 45–56.

[CIT0005] Mlenzana, N., 2020, ‘The role of virtual reality in South African physiotherapy practice’, *Journal of Rehabilitation Sciences* 35(4), 55–66.

[CIT0006] Motaze, N.V., Tumbo, J. & Mbombo, M., 2021, ‘Integration of physiotherapy services in South African public health facilities’, *BMC Health Services Research* 21, 115–129.33536017

[CIT0007] National Department of Health, 2019, *National Health Insurance bill*, Government Printer, Pretoria.

[CIT0008] SA Society of Physiotherapy, 2021, ‘Physiotherapy and the future: A strategic plan’, *SA Society of Physiotherapy Reports* 47(3), 5–10.

[CIT0003] Van Staden, D., 2021, ‘Investing in Health Professions Education in South Africa: A national development imperative’, *South African Journal of Higher Education* 35(1), 231–245. 10.20853/35-1-2916

[CIT0009] Winstein, C. & Requejo, P., 2015, ‘Innovative technologies for rehabilitation and health promotion: What is the evidence?’, *Physical Therapy* 95(3), 294–298. 10.2522/ptj.2015.95.2.294 25734191

